# Prevalence and correlates of active transportation to campus among Canadian post-secondary students: evidence from the Canadian Campus Wellbeing Survey

**DOI:** 10.3389/fspor.2026.1742348

**Published:** 2026-04-02

**Authors:** Lex Derkach, Meijun Hou, Caroline Wu, Guy Faulkner

**Affiliations:** School of Kinesiology, University of British Columbia, Vancouver, BC, Canada

**Keywords:** active travel, correlates, physical activity, university, young adults

## Abstract

**Introduction:**

Active transportation to campus may be an important source of physical activity while contributing to broader benefits to the environment. Currently, little is known about the transportation behaviour of post-secondary students. The aims of the current study were (a) to examine the prevalence of active transportation to campus in a national sample of post-secondary students, and (b) to examine the sociodemographic, contextual and lifestyle characteristics associated with those transportation behaviours.

**Methods:**

This large-sample cross-sectional analysis used self-reported data from the Canadian Campus Wellbeing Survey across three deployment time periods. Descriptive analyses were conducted to characterize the sample, mental health indicators, and prevalence of active transportation. Binary logistic regression was used to examine associations between sociodemographic, contextual, and mental health factors and transportation mode (active vs. not active). Results were reported as adjusted odds ratios with 95% confidence intervals. In total, 41,553 respondents were included in the sample from 56 post-secondary institutions (33 universities, 15 colleges, and 8 institutes).

**Results:**

Overall, 16.4% of respondents reported using an active mode of transportation to campus. Active transportation prevalence was highest among respondents aged 20–24 (20.6%; aOR = 1.37, 95% CI: 1.14–1.63, vs. under 20) and those who identified as a man (17.9%; aOR = 1.20, 95% CI: 1.07–1.35) or non-binary or Two-Spirit (19.7%; aOR = 1.44, 95% CI: 1.18–1.75), relative to women. Students from Ontario (22.1%) and Nova Scotia (23.6%) had the highest prevalence of active transportation; compared with Ontario, odds were lower in Alberta (aOR = 0.31, 95% CI: 0.17–0.56) and British Columbia (aOR = 0.29, 95% CI: 0.15–0.54). Commute time showed the strongest association: prevalence was 27.7% for 0–30 min, and odds were substantially lower for 31–60 min (aOR = 0.08, 95% CI: 0.06–0.12) and over 60 min (aOR = 0.01, 95% CI: 0.01–0.03), compared with 0–30 min. Students who met the Canadian physical activity guidelines (18.3%) reported higher prevalence of active transportation; not meeting guidelines was associated with lower odds (aOR = 0.62, 95% CI: 0.54–0.71).

**Conclusion:**

Most Canadian post-secondary students travel to their institution by transit or car. Correlates of active transportation were consistent with existing research. Students using active transportation were more likely to be younger, male and of higher SES. Active transportation was more likely to occur in larger, urban settings. They were also more likely to report meeting the Canadian physical activity guidelines. Future policy and practice should focus on how to support more students living within a reasonable distance from their campus to walk or cycle.

## 1 Introduction

Active transportation (AT) refers to non-motorized modes including walking, cycling and the use of human-powered or hybrid mobility aids such as wheelchairs, scooters, e-bikes, rollerblades, or even snowshoes. AT is well recognized for its many co-benefits that extend beyond physical activity, including safety, health, economic, environmental, transport quality, and social outcomes (1). Increased physical activity (PA) due to shifts to active transportation have been associated with substantial net health benefits (2). Environmentally, AT represents a sustainable form of transportation and shifts in mode choice from motorized vehicles to AT can lead to reductions in air pollution (3). AT also represents an inclusive and equitable form of transport, given that walking and cycling are inexpensive and more accessible in comparison to the use of motorized vehicles (4). Given these wide-ranging benefits, it has been proposed that active transportation has the potential to simultaneously address three global public health challenges of the 21st century—global climate change, air pollution, and physical inactivity (5).

There is a wide range of evidence related to active school travel and the socio-demographic factors and PA-levels for children and adolescents (6, 7). Although still limited, a growing body of literature on the travel behaviour of post-secondary students is now emerging (8, 9). Compared to the general population, post-secondary students are considered to have relevantly unique travel needs (8). For example, post-secondary students tend to make more trips and use public transit more frequently than the general population. While effective and efficient trips to and from the post-secondary campus are important, transportation systems and policies must also be designed to meet the equally significant non-academic needs of students to get to other locations for work, shopping, socializing and recreation (10). Over 2 million Canadians attend a post-secondary institution (11), and this represents a relatively large percentage of trip-makers whose impact will influence travel demand and travel behaviour in regions where institutions are located. Staff and student commuting to and from campus might even be the single largest impact a post-secondary institution has on the environment (12). As these students are mostly young adults, a particular interest in their travel behaviour during these post-secondary years has also been of interest due to the potential for the habits formed during this developmental stage to influence future travel behaviours (9). Overall, with the potential short- and long-term implications for the transportation system, it remains essential to understand the activity-travel behaviour of post-secondary students (13).

Research in this area, particularly in the Canadian context, has been hampered by underrepresentation of post-secondary students in transportation surveys. The 2019 StudentMoveTO survey addressed this gap through a large-scale post-secondary student travel survey conducted in the Greater Toronto and Hamilton Area (GTHA) during October and November 2019 (10). Four public colleges and six universities participated in the study representing nearly 328,000 students across 26 campuses located across the region in both urban and suburban environments. The survey collected information on individual and household socio-demographics, a one-day travel diary, typical transportation characteristics, monthly usage frequency of different types of transportation modes, and attitudes towards campus life, travel motivations, and subjective well-being. With an overall response rate of 6%, a total of 18,513 students participated in the survey (i.e., completed at least parts of the survey), thus making StudentMoveTO one of the largest and most diverse student data to be collected and analyzed (10). In terms of key findings, almost two thirds (60%) of all students reported using either local transit (buses, streetcars, and subways) or regional transit (GO Trains, GO buses) as their primary travel mode when travelling to campus. Walking (18%) and cycling (3%) were less common. Notably, students on average had a one-way travel distance of 14.6 kilometers and a travel duration of 45.9 min. In a further analysis of respondents who completed a travel diary, the majority (61%) of students reported transportation was a barrier to their campus participation and 30% perceived their transportation as a barrier to academic success (14).

The StudentMoveTO survey was restricted to one region which is the largest urban area in Canada. Travel mode beyond this region across Canada remains less known while little evidence is available related to the transportation behaviors to campus and its association with sociodemographic characteristics and physical activity (15). Therefore, the aims of the current study were (a) to examine the prevalence of active transportation to campus in a national sample of post-secondary students, and (b) to examine the sociodemographic, contextual and lifestyle characteristics associated with those transportation behaviors. Addressing these aims may inform future evidence-based planning and policy by urban and transportation planners, post-secondary institutions, and governments.

## 2 Materials and methods

### 2.1 Study design

We conducted a cross-sectional analysis using self-reported data from the Canadian Campus Wellbeing Survey (CCWS). The CCWS, an online questionnaire, was developed as a mechanism for monitoring health and well-being among Canadian post-secondary students. Detailed information about the CCWS study design, methods, survey measures and data access policy is available at https://www.ccws-becc.ca (16). Additional information about the survey measures, including on validity and reliability, is also available (17).

The survey was delivered online during three deployment windows: Fall 2023 (5), Winter 2024 (34), and Winter 2025 (18). In total, 56 post-secondary institutions participated, including one institution that took part in both Fall 2023 and Winter 2025. Institutions were located in Alberta, British Columbia, Manitoba, Nova Scotia, Ontario, and Quebec, and included 33 universities, 15 colleges, and eight institutes of varying sizes (small ≤ 5,000; medium 5,001–20,000; large ≥ 20,001 students). Recruitment strategies varied by institution, with most opting to survey their entire student body (census approach). The CCWS was delivered through Qualtrics, with students receiving email invitations and follow-up reminders throughout the survey window. Most institutions sent three reminders, and the overall median across all institutions was also three. The average length of the survey window was 27 days.

Across the 57 deployments, 751,824 students were invited to complete the online survey, of whom 78,711 responded and answered at least four questions, yielding an overall response rate of 10.5%. The mean institutional response rate was 16.6% (SD = 9.2). Among respondents, 63,435 reached the final page of the questionnaire (with some items possibly skipped), while 15,276 answered more than four questions but exited earlier.

Ethical approval was granted by the University of British Columbia Behavioural Research Ethics Board (H19-01907) and by participating institutions.

### 2.2 Participants and inclusion criteria

We analysed an existing CCWS dataset and applied inclusion criteria based on residence and transportation responses. Students were included if they lived off campus (with family, alone, or with roommates/friends), reported unstable housing, or selected “prefer not to answer” for housing status. Students living in university or college residences or other on-campus housing were excluded because transportation questions were not administered to them. Respondents who reported “Not applicable” for transportation time were also excluded. In addition, we restricted the analytical sample to students with complete data on all study variables. The final sample included 41,553 students, indicating that 37,158 of the 78,711 respondents who answered at least four questions were excluded due to missing data on one or more study variables. As a diagnostic check, patterns of missing data were assessed using Little's MCAR test, which indicated that missingness was not completely at random. Patterns of missingness indicated that included participants were more likely to be younger, full-time, and not employed, while differences by gender, SES, and mental health indicators were modest (see Supplementary Table S1), suggesting that selection bias, while possible, is unlikely to be driven primarily by mental health–related non-response. Differences between included and excluded respondents are described in Supplementary Table S1. Demographic characteristics of the analytic sample are presented in Tables 1, 2.

**Table 1:** Sample characteristics of sociodemographic factors among Canadian post-secondary students (*N* = 41,553).

Sample characteristic	Number of Participants/Mean	Proportion of sample %/SD
Age (yrs)	24.3	7.2
Age		
Under 20	8,809	21.2
20–24	19,851	47.8
25–29	6,058	14.6
30–34	2,953	7.1
Over 35	3,882	9.3
Gender		
Woman	27,492	66.2
Man	12,794	30.8
Non-binary person, Two-Spirit	1,267	3.0
Residency Status		
Domestic	32,345	77.8
International	9,208	22.2
Student Status		
Full-Time	38,015	91.5
Part-Time	3,538	8.5
Socioeconomic Status		
High	32,652	78.6
Low	8,901	21.4
Employment Status		
Employed	24,704	59.5
Unemployed	16,849	40.5
Financial Stress		
No financial stress at all	4,701	11.3
Very little financial stress	7,306	17.6
Some financial stress	11,711	28.2
A great deal of financial stress	9,191	22.1
Quite a bit of financial stress	8,644	20.8

Values represent the number and percentage of participants in each category, or the mean and standard deviation where applicable, based on the analytical sample (*N* = 41,553).

**Table 2:** Sample characteristics of contextual, mental health and behavioural factors among Canadian post-secondary students (*N* = 41,553).

Sample characteristic	Number of Participants	Proportion of sample %
CONTEXTUAL FACTORS		
Institution type		
College	8,411	20.2
Institute	3,167	7.6
University	29,975	72.1
Institution Size		
Small (5,000 or fewer)	3,832	9.2
Medium (5,001–20,000)	15,728	37.9
Large (20,001 or greater)	21,993	52.9
Province		
Alberta	8,738	21.0
British Columbia	5,380	12.9
Manitoba, Quebec	2,128	5.1
Nova Scotia	5,021	12.1
Ontario	20,286	48.8
Campus Location		
Rural	353	0.8
Urban	41,200	99.2
Survey Deployment Term		
Fall	2,663	6.4
Winter	38,890	93.6
Commute Time		
0–30 min	23,040	55.4
31–60 min	11,681	28.1
Over 60 min	6,832	16.4
Travel Mode		
Walk	6,389	15.4
Bicycle	414	1.0
Public transit	19,761	47.6
Vehicle (alone)	11,754	28.3
Vehicle (with others/carpool)	3,235	7.8
Transportation Type (Grouped)		
Active	6,803	16.4
Not Active	34,750	83.6
MENTAL HEALTH AND BEHAVIORAL FACTORS		
Mental Wellbeing (WEMWBS)		
Average Mental Wellbeing	25,085	60.4
High Mental Wellbeing	3,945	9.5
Low Mental Wellbeing	12,523	30.1
Psychological Distress (K10)		
Little/no mental distress	9,658	23.2
Mild mental distress	8,791	21.2
Moderate mental distress	8,770	21.1
Severe mental distress	14,334	34.5
Physical Activity		
Meeting Canadian PA guidelines	28,508	68.6
Not meeting Canadian PA guidelines	13,045	31.4

Values represent the number and percentage of participants in each category based on the analytical sample (*N* = 41,553).

### 2.3 Outcome measure

The outcome variable was transportation mode. Students were asked to indicate their usual mode of transportation to campus. Responses were recoded into two categories: active transportation (walking or cycling) and not active transportation (personal vehicle, carpool, or public transit). A binary variable was then created (1 = active transportation, 0 = not active transportation).

### 2.4 Sociodemographic factors

#### 2.4.1 Age

Students reported their age in years. For analysis, we created categorical groups: under 20, 20–24, 25–29, 30–34, and 35 years and over. For regression analyses, students under 20 years of age were used as the reference category.

#### 2.4.2 Gender

Students were asked to identify their gender as woman (reference), man, non-binary person, or Two-Spirit. For analysis, responses of non-binary and Two-Spirit were combined into a single category. “I prefer not to answer” was treated as missing.

#### 2.4.3 Residency status

Institutions provided cohort information on whether students were registered as domestic or international. For analysis, domestic students served as the reference category.

#### 2.4.4 Student status

Institutions also provided information on whether students were enrolled full time (reference) or part time.

#### 2.4.5 Socioeconomic status (SES)

As a proxy for SES, students were asked about the highest level of formal education completed by their parent(s) or guardian(s). Responses were classified as low SES if the highest level reported was high school or less, and high SES (reference) if the parent(s) had completed a college program, university degree, or graduate/professional degree. Responses of “I don't know,” “Prefer not to answer,” or “Not applicable” were treated as missing.

#### 2.4.6 Employment status

Employment was assessed by asking students the average number of hours of paid work per week during the school year. Students reporting at least one hour of paid work were considered employed (reference), while those reporting zero hours were classified as unemployed.

#### 2.4.7 Financial stress

Financial stress was measured with a single item: “To what extent, if at all, have you experienced financial stress due to your tuition and living expenses while at your current post-secondary institution?” Responses were provided on a 5-point scale from *no financial stress at all* to *a great deal of financial stress*. For regression analyses, *no financial stress at all* served as the reference category.

### 2.5 Contextual factors

#### 2.5.1 Institution type

Institutions were classified as universities, colleges, or institutes. Universities were used as the reference category.

#### 2.5.2 Institution size

Total student enrolment provided by each institution was grouped into three categories: small (≤5,000 students), medium (5,001–20,000 students), and large (≥20,001 students). Small institutions were used as the reference category.

#### 2.5.3 Province

Institutions were grouped by geographic region. Categories included Ontario (reference), Alberta, British Columbia, and Nova Scotia, with Manitoba and Quebec combined into a single category to preserve institutional confidentiality.

#### 2.5.4 Campus location

Campus locations were coded as urban or rural using campus postal codes supplied by participating post-secondary institutions. When a student-campus postal code was not available, the institution's main campus postal code was used. Classification followed Canada Post's Forward Sortation Area (FSA) logic: postal codes with a “0” as the second character are designated as rural delivery areas, while those with a value from 1 to 9 in the second position are classified as urban delivery areas (18). In regression analyses, urban campuses were used as the reference category.

#### 2.5.5 Commute time

Commute time was assessed with the item: “*How long is your commute to campus (one-way)?”* Response options were *0–30 min*, *31–60 min*, *Over 60 min*, and *Not applicable*. For analysis, a categorical variable was created with three groups (0–30 min, 31–60 min, and over 60 min). Students who selected *Not applicable* were excluded from the analytical sample. In regression analyses, the 0–30 min category served as the reference group.

#### 2.5.6 Deployment term

The CCWS was administered during either the Fall or Winter academic term. Fall deployments were conducted between September and December, while Winter deployments were conducted between January and May. In regression analyses, the Fall term served as the reference category.

### 2.6 Mental health and behavioural factors

#### 2.6.1 Mental wellbeing (WEMWBS)

Mental wellbeing was measured using the 14-item Warwick–Edinburgh Mental Wellbeing Scale (WEMWBS) (19), which assesses positive psychological functioning and subjective wellbeing over the preceding two weeks. Items are rated on a 5-point Likert scale from 1 (*none of the time*) to 5 (*all of the time*) and summed to produce a total score ranging from 14 to 70, with higher values reflecting greater wellbeing. For analysis, scores were categorized as low (≤40; reference), average (41–58), and high (59–70).

#### 2.6.2 Psychological distress (K10)

Psychological distress was measured using the 10-item Kessler Psychological Distress Scale (K10) (20), which captures symptoms of anxiety and depression over the past month. Items are rated on a 5-point scale from 1 (*none of the time*) to 5 (*all of the time*) and summed to yield a total score ranging from 10 to 50, with higher values reflecting greater distress. For analysis, scores were categorized as little or no distress (<20; reference), mild (20–24), moderate (25–29), and severe (30–50).

#### 2.6.3 Physical activity

Physical activity was assessed using the International Physical Activity Questionnaire (IPAQ) (21). The IPAQ includes four items that capture the frequency (days) and duration (hours and minutes) of their moderate and vigorous physical activity (MVPA) performed over the last seven days. In line with the Canadian 24-Hour Movement Guidelines (22), students reporting more than 150 min per week were categorized as meeting physical activity guidelines (reference category), while those reporting fewer than 150 min per week were categorized as not meeting guidelines.

### 2.7 Statistical analysis

We conducted descriptive analyses to characterize the sample, mental health indicators, and prevalence of active transportation. We then used binary logistic regression to examine associations between sociodemographic, contextual, and mental health factors and transportation mode (active vs. not active). Results are reported as adjusted odds ratios (aORs) with 95% confidence intervals (CIs). The adjustment strategy was intended to describe associations rather than estimate causal effects. To assess the potential impact of selection bias on our primary findings, we conducted a sensitivity analysis using Inverse Probability Weighting (IPW). Weights were derived from a logistic regression model predicting study inclusion based on sociodemographic characteristics with complete data (age, gender, residency, student status, employment, SES, and institution type). We then re-estimated the prevalence and adjusted odds ratios using weights to explore if the findings were representative of the initial sample (see Supplementary Table S2). We accounted for clustering at the institutional level by estimating cluster-robust standard errors. Statistical significance was set at *p* < 0.05. All analyses were conducted in Python version 3.13.1 using the pandas (v2.2.3), statsmodels (v0.14.4), and numpy (v2.2.2) libraries.

## 3 Results

Overall, 16.4% of post-secondary students reported AT to campus. Table 3 presents the prevalence of active transportation by sociodemographic, contextual, and health-related factors, along with adjusted odds ratios (aORs) and 95% confidence intervals (CIs).

**Table 3:** Active transportation prevalence and associations with sociodemographic, contextual, and mental health and behavioural factors.

Sample characteristic	Active prevalence (95% CI)	Adjusted odds ratio (95% CI)	*p* value
Age group (yrs)			
Under 20	13.1 (12.4–13.8)	1 (reference)	
20–24	20.6 (20.0–21.1)	1.37 (1.14–1.63)	**0**.**001**
25–29	15.1 (14.2–16.0)	1.14 (0.83–1.55)	0.424
30–34	11.7 (10.6–12.9)	0.91 (0.66–1.25)	0.555
Over 35	7.9 (7.1–8.7)	0.74 (0.49–1.12)	0.156
Gender			
Woman	15.5 (15.1–15.9)	1 (reference)	
Man	17.9 (17.3–18.6)	1.2 (1.07–1.35)	**0**.**002**
Non-binary person, Two-Spirit	19.7 (17.6–22.0)	1.44 (1.18–1.75)	**<0**.**001**
Residency status			
Domestic	16.3 (15.9–16.7)	1 (reference)	
International	16.7 (16.0–17.5)	1.4 (0.98–2.01)	0.063
Student status			
Full-Time	16.9 (16.5–17.3)	1 (reference)	
Part-Time	10.6 (9.6–11.6)	0.81 (0.65–1.01)	0.057
Socioeconomic status			
High	17.8 (17.4–18.3)	1 (reference)	
Low	11 (10.4–11.7)	0.71 (0.63–0.82)	**<0**.**001**
Employment status			
Employed	13.9 (13.5–14.4)	1 (reference)	
Unemployed	20 (19.4–20.6)	1.4 (1.26–1.56)	**<0**.**001**
Financial Stress			
No financial stress at all	16.9 (15.9–18.0)	1 (reference)	
Very little financial stress	19.5 (18.6–20.4)	1.12 (0.98–1.29)	0.098
Some financial stress	17.2 (16.5–17.9)	1.1 (0.91–1.32)	0.324
Quite a bit of financial stress	15 (14.2–15.7)	1.01 (0.83–1.24)	0.902
A great deal of financial stress	13.9 (13.2–14.6)	1.08 (0.86–1.35)	0.504
Institution type			
College	6.9 (6.4–7.4)	1 (reference)	
Institute	6.8 (6.0–7.8)	1.45 (0.55–3.87)	0.453
University	20 (19.6–20.5)	1.83 (0.77–4.34)	0.172
Institution Size			
Small (5,000 or fewer)	14.8 (13.7–16.0)	1 (reference)	
Medium (5,001–20,000)	11.7 (11.2–12.2)	0.48 (0.25–0.89)	**0**.**021**
Large (20,001 or greater)	20 (19.5–20.5)	1.33 (0.66–2.67)	0.429
Province			
Ontario	22.1 (21.6–22.7)	1 (reference)	
Alberta	7.1 (6.6–7.7)	0.31 (0.17–0.56)	**<0**.**001**
British Columbia	7.9 (7.2–8.6)	0.29 (0.15–0.54)	**<0**.**001**
Manitoba, Quebec	4.1 (3.3–5.0)	0.42 (0.11–1.52)	0.185
Nova Scotia	23.6 (22.4–24.8)	2.2 (0.94–5.19)	0.07
Campus Location			
Urban	16.4 (16.1–16.8)	1 (reference)	
Rural	7.9 (5.5–11.2)	0.93 (0.54–1.59)	0.786
Commute Time			
0–30 min	27.7 (27.2–28.3)	1 (reference)	
31–60 min	3.2 (2.9–3.5)	0.08 (0.06–0.12)	**<0**.**001**
Over 60 min	0.6 (0.5–0.9)	0.01 (0.01–0.03)	**<0**.**001**
Survey Deployment Term			
Winter	17 (16.6–17.4)	1 (reference)	
Fall	7.3 (6.4–8.3)	0.55 (0.27–1.13)	0.104
Mental Wellbeing (WEMWBS)			
Low Mental Wellbeing	15.7 (15.1–16.4)	1 (reference)	
Average Mental Wellbeing	17.3 (16.8–17.7)	0.96 (0.87–1.07)	0.467
High Mental Wellbeing	12.8 (11.7–13.8)	0.77 (0.61–0.97)	**0**.**026**
Psychological Distress			
Little/no mental distress	16.5 (15.8–17.3)	1 (reference)	
Mild mental distress	17.6 (16.8–18.4)	1.04 (0.96–1.12)	0.389
Moderate mental distress	17 (16.2–17.8)	0.99 (0.91–1.09)	0.871
Severe mental distress	15.1 (14.5–15.7)	0.92 (0.84–1.01)	0.089
Physical Activity			
Meeting Canadian PA guidelines	18.3 (17.9–18.8)	1 (reference)	
Not meeting Canadian PA guidelines	12.1 (11.6–12.7)	0.62 (0.54–0.71)	**<0**.**001**

Values represent prevalence estimates with corresponding 95% confidence intervals (CIs) and adjusted odds ratios (aORs) estimated from a multivariable binary logistic regression model including age group, gender, residency status, student status, socioeconomic status, employment status, financial stress, institution type and size, province, campus location, commute time, survey deployment term, mental wellbeing, psychological distress, and physical activity. Ninety-five percent confidence intervals for aORs were computed using institution-level cluster-robust standard errors.

Bold values indicate significant at *p* < .05.

### 3.1 Sociodemographic factors

#### 3.1.1 Age

Students aged 20–24 years had the highest prevalence of active transportation (20.6%), with 37% higher odds compared with those under 20 (aOR = 1.37, 95% CI: 1.14–1.63, *p* = 0.001). Prevalence declined with age, reaching 7.9% among those aged 35 or older. However, differences beyond age 24 were not statistically significant.

#### 3.1.2 Gender

Men (17.9%) and students identifying as non-binary or Two-Spirit (19.7%) reported a higher prevalence of active transportation compared with women (15.5%). In adjusted models, men had 20% higher odds of active transportation (aOR = 1.20, 95% CI: 1.07–1.35, *p* = 0.002), and non-binary or Two-Spirit students had 44% higher odds (aOR = 1.44, 95% CI: 1.18–1.75, *p* *<* 0.001), relative to women.

#### 3.1.3 Socioeconomic status

Students from low SES households were less likely to report AT (11.0%) compared with those from high SES households (17.8%), with 29% lower odds (aOR = 0.71, 95% CI: 0.63–0.82, *p* < 0.001).

#### 3.1.4 Employment status

Unemployed students reported higher prevalence of active transportation (20.1%) than employed students (13.9%), with 41% higher odds (aOR = 1.41, 95% CI: 1.27–1.57, *p* < 0.001).

Other sociodemographic factors, including student status, residency status, and financial stress, were not significantly associated with active transportation.

### 3.2 Contextual factors

#### 3.2.1 Province

Marked provincial differences were observed. Students in Ontario (22.1%) and Nova Scotia (23.6%) reported the highest prevalence of active transportation, whereas students in Alberta (7.1%) and British Columbia (7.9%) reported the lowest. Compared with Ontario, the odds of active transportation were substantially lower in Alberta (aOR = 0.31, 95% CI: 0.17–0.56, *p* < 0.001) and British Columbia (aOR = 0.29, 95% CI: 0.15–0.54, *p* < 0.001).

#### 3.2.2 Institution size

 Students at medium-sized institutions (11.7%) were less likely to report AT than those at small institutions (14.8%), with 52% lower odds (aOR = 0.48, 95% CI: 0.25–0.89, *p* = 0.021).

#### 3.2.3 Commute time

Commute duration showed the strongest association. Among students with short commute times (0–30 min), 27.7% reported active transportation. Prevalence fell to 3.2% among those travelling 31–60 min and to 0.6% among those with commutes over 60 min. Compared with short commutes, odds of active transportation were reduced by 92% for 31–60 min (aOR = 0.08, 95% CI: 0.06–0.12, *p* < 0.001) and by 99% for commutes longer than 60 min (aOR = 0.01, 95% CI: 0.01–0.03, *p* < 0.001).

Campus location and deployment term were not significantly associated with active transportation.

### 3.3 Mental health and behavioural factors

#### 3.3.1 Mental wellbeing

Students with high mental wellbeing reported lower prevalence of active transportation (12.8%) compared with those with low wellbeing (15.7%), corresponding to 23% lower odds (aOR = 0.77, 95% CI: 0.61–0.97, *p* = 0.026). Psychological distress was not significantly associated with active transportation.

#### 3.3.2 Physical activity

Students meeting Canadian physical activity guidelines reported higher prevalence of active transportation than those not meeting the guidelines (18.3% vs. 12.1%). Not meeting the guidelines was associated with 38% lower odds of active transportation (aOR = 0.62, 95% CI: 0.54–0.71, *p* < 0.001).

#### 3.3.3 Sensitivity analysis

The results of the IPW sensitivity analysis were largely consistent with the primary unweighted analysis (see Supplementary Table S2). Notably, the associations between active transportation and residency status, student status, institution type, and survey deployment term, which were non-significant in the primary analysis, became statistically significant after weighting for selection bias. Specifically, international students had 42% higher odds of using active transportation compared to domestic students (wOR = 1.42, 95% CI: 1.31–1.54, *p* < 0.001), while part-time students had 20% lower odds compared to full-time students (wOR = 0.80, 95% CI: 0.70–0.91, *p* < 0.001). Regarding institution type, students attending institutes (wOR = 1.49, 95% CI: 1.22–1.81, *p* < 0.001) and universities (wOR = 1.81, 95% CI: 1.60–2.03, *p* < 0.001) reported higher odds of active transportation than college students. Fall term deployment was associated with 44% lower odds of active transportation compared to Winter (wOR = 0.56, 95% CI: 0.47–0.68, *p* < 0.001). These findings suggest that selection bias in the unweighted sample may have partially masked the strength of these associations.

## 4 Discussion

The first aim of the current study was to examine the prevalence of active transportation to campus in a Canadian sample of post-secondary students. Overall, public transit was the most common mode of transportation (47.6%), followed by travelling by vehicle, either alone or with others (36.1%). Active transportation accounted for 16.4% of students, with cycling being relatively uncommon (1%). These findings are broadly consistent with existing research suggesting that passive means of transport are the most commonly used to travel to university (10, 15, 23, 24). While the majority of students in our sample do not report AT to campus there remains some variation based on a range of sociodemographic, contextual and lifestyle characteristics addressing our second aim.

Based on the literature, different types of factors affect students' transportation patterns (13). These include (1) individual and household socio-demographic characteristics (e.g., age, gender, income, driving license ownership, vehicle ownership, household size); (2) built environment attributes (e.g., population density, land use mix, road and sidewalk densities); (3) trip characteristics and level-of-service attributes (e.g., travel distance, mode-specific travel time and cost); and (4) attitudes and preferences which are not assessed in the CCWS.

In terms of socio-demographic characteristics, the most consistent predictors of active transportation were age, gender, SES, and employment status. Recent Canadian population surveys also confirm that younger adults and men are more likely to use active travel for getting to and from places compared to older adults and women (25). In terms of gender, disparities in active travel typically seen in school settings (26) are extended in the current research into early adulthood. As others have noted, social and cultural norms likely contribute to these disparities while gendered differences in barriers to active transportation (e.g., concerns about safety and harassment (15) may be particularly salient.

It is likely that SES and employment status interact. Employed and lower SES students were less likely to actively travel. The campus is only one destination of potentially many for post-secondary students (10) and students who are employed may need greater travel flexibility for navigating home, work and school destinations. Conversely, higher SES students may have less need for employment during their studies. Such reasoning is speculative and may also mask important differences in distances between residential location and one's institution. Higher SES may also indicate capacity to live closer to that institution making active transportation more feasible.

This potential for spatial heterogeneity in active transportation rates is clearer when considering the potential influence of built environment and trip characteristics. Not surprisingly, the strongest correlate of active transportation was commute time. Beyond a self-reported time of 30 min, the odds of active transportation were negligible. Although commute time does not necessarily directly reflect distance (e.g., congestion; transit transfer time), this factor is consistently and negatively associated with active school travel (27). Such shorter commute times are likely more feasible in large, urban, and high-density settings which are more likely to have transit and pedestrian/cycling infrastructure. Accordingly, higher active transportation rates were seen at universities compared to colleges, larger compared to small and medium sized institutions, and urban vs. rural institutions. Active transportation rates were highest in Ontario and Nova Scotia. Notably, active transportation rates in Ontario (22.1%) were similar to that reported (21%) pre-COVID in the 2019 StudentMoveTO survey (10). Active transportation in this sample was more common for students who lived in older or redeveloped neighbourhoods with high land use mix and easy access to transit services and bicycle infrastructure (9). Higher active transportation rates in Nova Scotia are also not unexpected. This geographically small province has a high concentration of post-secondary institutions relative to its population of approximately 1 million.

Our findings also add to the limited evidence base that active transportation to post-secondary education is associated with young adults achieving physical activity guidelines. In a sample of Chilean University students (23), higher physical activity was reported by those using more active modes of transportation compared to those using transit to university. Notably, when this association was analyzed based on those living shorter, medium, or further distances from university, the association only remained for those living a medium distance (i.e., from 2 km to 5 km). Without more detail about distance and mode, it is not possible to discern the relative contribution of active transportation to overall physical activity levels among the CCWS sample.

Associations between travel mode and measures of mental health were unclear. While there was no association with psychological distress, there was a modest difference in that students reporting high mental well-being were less likely to report AT to campus compared to students with low well-being. Walking or biking have been associated with higher travel satisfaction (and consequently higher subjective well-being) among GTHA post-secondary students (14). As these authors examined, it is possible that a relationship between transportation mode and well-being can only be considered through the interplay of other factors that may impact course selection, capacity to participate in on-campus extra-and co-curricular activities, and overall perceived academic success.

There are a range of important implications of this study for policy and practice. Distance and related transportation times will remain a significant barrier to the many young adults attending Canadian post-secondary institutions. At a population level, there may be thresholds at which walking or cycling for active travel are considered feasible with some reports suggesting average walking distances between 1.1 and 1.5 kms and average cycling distances between 3.9 and 5 kms (28, 29). As our results indicate, supporting students who live within 30 min of campus to use transit and/or active travel is warranted. Educational campaigns and interventions to promote the co-benefits of active transportation (30) that are gender-sensitized as well as further developing supportive pedestrian and cycling infrastructure may be a start. Bike share programs also have a role to play (e.g., https://planning.ubc.ca/bikeshare) in supporting greater cycling.

An important strategy is increasing the supply of affordable student housing near campus to encourage the use of sustainable transportation modes like walking and cycling (13). Municipal and provincial planners can also contribute by using or developing policy tools to support affordable student housing near campuses by amending zoning by-laws or land use policies where appropriate (14). Finally, public transit is the most common mode to campus which reinforces continued efforts to subsidise transit passes where possible and increasing transit services, express routes to campus, and infrastructure improvements to increase reliability such as bus-only lanes on key routes (14). Such initiatives may also encourage a mode shift from vehicle travel to transit.

## 5 Strengths and limitations

The large sample accounting for institutional clustering, with representation from several Canadian provinces, are strengths of the study as is the inclusion of a number of validated measures of relevant covariates including physical activity and mental health. Several limitations should be acknowledged in our study. First, the cross-sectional design utilized in our research restricts our ability to determine the directionality of the observed associations and limits our ability to make causal inferences. Second, results are based on self-reported data which are subject to social desirability and response bias. Third, although the sample size is larger than existing Canadian studies examining post-secondary travel mode, sampling for the CCWS is not designed to be nationally representative. Moreover, Canadian seasonal variations were not controlled for in this study, as institutions deployed the survey at different points within the same academic term. Future research would benefit from incorporating more granular temporal and climate data to better account for seasonal and regional variability in AT behaviours. Fourth, the analytical sample did not include students living on campus, defined as those residing in university or college residences or other on-campus housing, because transportation questions were not administered to these groups. This exclusion and reliance on respondents with complete data only limits the generalizability of findings to the broader student population. On-campus students are likely to engage in short active trips, such as walking or cycling between residences and campus facilities, which were not captured in this analysis. Consequently, the overall prevalence of active transportation may underestimate total levels of active travel among all students. Fifth, there is the potential for non-differential exposure misclassification. In many instances, for example, public transit may involve physical activity (e.g., walking to and from stops), and grouping transit with private vehicle use may attenuate observed associations. Given our research questions and the nature of the CCWS which is not designed as a transportation survey, transit modes were dichotimized for analytic interpretation.

Finally, interpretation of SES for international students should be approached carefully. The indicators used in this study, such as parental education as a proxy for SES, are grounded in Canadian contexts and may more accurately reflect the circumstances of domestic students. For international students, these measures may not capture their lived realities, since educational attainment and financial resources in their country of origin do not necessarily correspond to socioeconomic position in Canada. This limitation suggests that SES classifications may be less applicable for international students, which could influence the observed associations.

## 6 Conclusion

Most Canadian post-secondary students travel to their institution by transit or car. Correlates of active transportation were consistent with existing research. For example, students reporting AT were more likely to be younger, male and of higher SES. Active transportation was more likely to occur in larger, urban settings. They were also more likely to report meeting the Canadian physical activity guidelines. Future policy and practice should focus on how to support more students living within a reasonable distance from their campus to walk or cycle.

## Data Availability

The raw data supporting the conclusions of this article will be made available by the authors, without undue reservation.
