# Supplementary material for: Prevalence and correlates of active transportation to campus among Canadian post-secondary students: evidence from the Canadian Campus Wellbeing Survey
Source: Front Sports Act Living. 2026 Apr 2;8:1742348. doi: 10.3389/fspor.2026.1742348 (PMC13083187; doi:10.3389/fspor.2026.1742348)
Supplement: Supplementary file 1 [file Datasheet1.pdf]

Supplementary

Supplementary Table S1. Descriptive characteristics of included versus excluded respondents

	**Included N**	**Included %**	**Excluded N**	**Excluded %**	**Difference (Included − Excluded), pp**
**Age**					
Under 20	8809	21.2	2054	16.9	+4.3
20-24	19851	47.8	4329	35.7	+12.1
25-29	6058	14.6	1901	15.7	-1.1
30-34	2953	7.1	1257	10.4	-3.2
Over 35	3882	9.3	2598	21.4	-12.1
Missing	0	0.0	16179		
**Gender**					
Woman	27492	66.2	7332	66.9	-0.7
Man	12794	30.8	3369	30.7	+0.1
Non-binary person, Two-Spirit	1267	3.0	266	2.4	+0.6
Missing	0	0.0	17351		
**Residency Status**					
Domestic	32345	77.8	20773	73.4	+4.4
International	9208	22.2	7515	26.6	-4.4
Missing	0	0.0	30		
**Student Status**					
Full-Time	38015	91.5	24082	85.2	+6.3
Part-Time	3538	8.5	4186	14.8	-6.3
Missing	0	0.0	50		
**Socioeconomic Status**					
High	32652	78.6	6512	75.5	+3.0
Low	8901	21.4	2109	24.5	-3.0
Missing	0	0.0	19697		
**Employment Status**					
Employed	24704	59.5	6617	65.1	-5.7
Unemployed	16849	40.5	3545	34.9	+5.7
Missing	0	0.0	18156		
**Financial Stress**					
No financial stress at all	4701	11.3	1462	11.8	-0.5

Very little financial stress	7306	17.6	1974	16.0	+1.6
Some financial stress	11711	28.2	3582	29.0	-0.8
Quite a bit of financial stress	8644	20.8	2461	19.9	+0.9
A great deal of financial stress	9191	22.1	2862	23.2	-1.1
Missing	0	0.0	15977		
**Institution type**					
College	8411	20.2	6536	23.1	-2.8
Institute	3167	7.6	2547	9.0	-1.4
University	29975	72.1	19235	67.9	+4.2
**Institution Size**					
Small (5,000 or fewer)	3832	9.2	2953	10.4	-1.2
Medium (5,001 – 20,000)	15728	37.9	9667	34.1	+3.7
Large (20,001 or greater)	21993	52.9	15698	55.4	-2.5
**Province**					
Alberta	8738	21.0	6560	23.2	-2.1
British Columbia	5380	12.9	3793	13.4	-0.4
Manitoba, Quebec	2128	5.1	1115	3.9	+1.2
Nova Scotia	5021	12.1	3345	11.8	+0.3
Ontario	20286	48.8	13505	47.7	+1.1
**Campus Location**					
Rural	353	0.8	276	1.0	-0.1
Urban	41200	99.2	28030	99.0	+0.1
Missing	0	0.0	12		
**Survey Deployment Term**					
Fall	2663	6.4	1755	6.2	+0.2
Winter	38890	93.6	26563	93.8	-0.2
**Commute Time**					
0-30 minutes	23040	55.4	4337	47.1	+8.4
31-60 minutes	11681	28.1	2718	29.5	-1.4
Over 60 minutes	6832	16.4	2155	23.4	-7.0
Missing	0	0.0	19108		
**Travel Mode**					
Walk	6389	15.4	1089	11.9	+3.5
Bicycle	414	1.0	91	1.0	+0.0
Public transit	19761	47.6	4673	51.1	-3.6
Vehicle (alone)	11754	28.3	1994	21.8	+6.5
Vehicle (with others/carpool)	3235	7.8	651	7.1	+0.7
Other (please specify):	0	0.0	644	7.0	-7.0

Missing	0	0.0	19176		
**Transportation Type (Grouped)**					
Active	6803	16.4	1180	13.9	+2.5
Not Active	34750	83.6	7318	86.1	-2.5
Missing	0	0.0	19820		
**Mental Wellbeing (WEMWBS)**					
Average Mental Wellbeing	25085	60.4	12481	58.9	+1.4
High Mental Wellbeing	3945	9.5	2817	13.3	-3.8
Low Mental Wellbeing	12523	30.1	5876	27.8	+2.4
Missing	0	0.0	7144		
**Psychological Distress (K10)**					
Little/no mental distress	9658	23.2	4455	25.2	-2.0
Mild mental distress	8791	21.2	3506	19.9	+1.3
Moderate mental distress	8770	21.1	3563	20.2	+0.9
Severe mental distress	14334	34.5	6138	34.8	-0.3
Missing	0	0.0	10656		
**Physical Activity**					
Meeting Canadian PA guidelines	28508	68.6	9333	64.4	+4.3
Not meeting Canadian PA guidelines	13045	31.4	5170	35.6	-4.3
Missing	0	0.0	13815		

Note: This comparison is restricted to off-campus students, as transportation questions were not administered to students living in on-campus residences. Percentages for the excluded group are calculated among respondents with non-missing data for the given variable; missing values are shown but excluded from percentage denominators.

Table S2. Weighted active transportation prevalence and associations with sociodemographic, contextual, and mental health and behavioural factors.

	Weighted Active prevalence (95%CI)	Weighted odds ratio (95%CI)	*p* value
**Age group (yrs)**			
Under 20	13.0 (12.3-13.7)	1 (reference)	
20-24	20.5 (19.9-21.0)	1.37 (1.27-1.48)	**<0.001**
25-29	14.9 (14.0-15.8	1.12 (1.01-1.25)	**0.03**
30-34	11.5 (10.4-12.7)	0.91 (0.78-1.06)	0.22
Over 35	7.7 (6.9-8.6)	0.74 (0.63-0.87)	**<0.001**

**Gender**			
Woman	15.0 (14.6-15.5)	1 (reference)	
Man	17.6 (16.9-18.2)	1.20 (1.13-1.29)	**<0.001**
Non-binary person, Two-Spirit	19.8 (17.6-22.1)	1.50 (1.25-1.78)	**<0.001**
**Residency status**			
Domestic	15.8 (15.4-16.2)	1 (reference)	
International	16.5 (15.8-17.3)	1.42 (1.31-1.54)	**<0.001**
**Student status**			
Full-Time	16.7 (16.3-17.1)	1 (reference)	
Part-Time	10.0 (9.6-11.6)	0.80 (0.70-0.91)	**0.001**
**Socioeconomic status**			
High	17.4 (17.0-17.8)	1 (reference)	
Low	10.7 (10.0-11.3)	0.71 (0.66-0.77)	**<0.001**
**Employment status**			
Employed	13.6 (13.2-14.0)	1 (reference)	
Unemployed	19.6 (19.0-20.2)	1.40 (1.32-1.49)	**<0.001**
**Financial Stress**			
No financial stress at all	16.5 (15.5-17.6)	1 (reference)	
Very little financial stress	19.1 (18.2-20.0)	1.12 (1.01-1.24)	**0.04**
Some financial stress	16.7 (16.0-17.3)	1.09 (0.98-1.20)	0.11
Quite a bit of financial stress	14.5 (13.8-15.3)	1.00 (0.90-1.12)	0.98
A great deal of financial stress	13.6 (12.9-14.3)	1.08 (0.96-1.21)	0.19
**Institution type**			
College	6.8 (6.3-7.4)	1 (reference)	
Institute	6.9 (6.1-7.9)	1.49 (1.22-1.81)	**<0.001**
University	19.5 (19.1-20.0)	1.81 (1.60-2.03)	**<0.001**
**Institution Size**			
Small (5,000 or fewer)	14.1 (13.1-15.3)	1 (reference)	
Medium (5,001 – 20,000)	11.5 (11.0-12.0)	0.49 (0.43-0.56)	**<0.001**
Large (20,001 or greater)	19.4 (18.9-20.0)	1.34 (1.18-1.51)	**<0.001**
**Province**			
Ontario	21.6 (21.1-22.2)	1 (reference)	
Alberta	7.1 (6.6-7.7)	0.32 (0.29-0.35)	**<0.001**
British Columbia	7.7 (7.0-8.5)	0.30 (0.27-0.34)	**<0.001**
Manitoba, Quebec	4.1 (3.3-5.0)	0.40 (0.31-0.52)	**<0.001**
Nova Scotia	22.9 (21.8-24.1)	2.14 (1.93-2.37)	**<0.001**
**Campus Location**			
Urban	16.1 (15.7-16.4)	1 (reference)	
Rural	7.5 (5.2-10.7)	0.88 (0.58-1.34)	0.56
**Commute Time**			
0-30 minutes	27.0 (26.5-27.6)	1 (reference)	
31-60 minutes	3.2 (2.9-3.5)	0.09 (0.08-0.10)	**<0.001**
Over 60 minutes	0.7 (0.5-0.9)	0.02 (0.01-0.02)	**<0.001**
**Survey Deployment Term**			

Winter	16.6 (16.2-16.9)	1 (reference)	
Fall	7.2 (6.3-8.3)	0.56 (0.47-0.68)	**<0.001**
**Mental Wellbeing (WEMWBS)**			
Low Mental Wellbeing	15.4 (14.8-16.0)	1 (reference)	
Average Mental Wellbeing	16.8 (16.3-17.3)	0.96 (0.89-1.04)	0.32
High Mental Wellbeing	12.3 (11.3-13.4)	0.76 (0.66-0.88)	**<0.001**
**Psychological Distress**			
Little/no mental distress	15.9 (15.2-16.7)	1 (reference)	
Mild mental distress	17.1 (16.3-17.9)	1.03 (0.94-1.13)	0.50
Moderate mental distress	16.7 (15.9-17.4)	1.00 (0.91-1.10)	0.97
Severe mental distress	14.8 (14.2-15.4)	0.92 (0.83-1.02)	0.11
**Physical Activity**			
Meeting Canadian PA guidelines	18.3 (17.5-18.4)	1 (reference)	
Not meeting Canadian PA guidelines	11.7 (11.2-12.3)	0.62 (0.58-0.66)	**<0.001**

Note: Values represent prevalence estimates with corresponding 95% confidence intervals (CIs) and adjusted odds ratios (aORs) estimated from a multivariable binary logistic regression model including age group, gender, residency status, student status, socioeconomic status, employment status, financial stress, institution type and size, province, campus location, commute time, survey deployment term, mental wellbeing, psychological distress, and physical activity. Ninety-five percent confidence intervals for aORs were computed using institution-level cluster-robust standard errors
